# Effect of Intra-Workout Protein–Carbohydrate Co-Ingestion Versus Isocaloric Carbohydrate During Resistance Training on Muscle Fibre Hypertrophy and Oxidative Capacities in Young Men: A Randomized Controlled Trial

**DOI:** 10.3390/nu18142307

**Published:** 2026-07-14

**Authors:** Michael Svensson, Per Stål, Magnus Högström, Linda Nyström Hagfors, Ji-Guo Yu, Torbjörn Åkerfeldt, Andreas Isaksson

**Affiliations:** 1 Department of Community Medicine and Rehabilitation, Section of Sports Medicine, and Umeå School of Sport Sciences, Umeå University, 901 87 Umeå, Sweden; jiguo.yu@umu.se (J.-G.Y.); a_isaksson@live.se (A.I.); 2 Department of Integrative Medical Biology, Laboratory of Muscle Biology, Umeå University, 901 87 Umeå, Sweden; per.stal@umu.se; 3 Department of Surgical and Perioperative Sciences, Orthopaedics, Umeå University, 901 87 Umeå, Sweden; mphogstrom@gmail.com; 4 Department of Food, Nutrition and Culinary Science, Umeå University, 901 87 Umeå, Sweden; linda.hagfors@umu.se; 5 Department of Medical Sciences, Clinical Chemistry, Uppsala University, 751 85 Uppsala, Sweden; torbjorn.akerfeldt@akademiska.se

**Keywords:** muscle biopsy, myosin heavy chain, nutrient timing, capillarization, IGF-1, body composition

## Abstract

**Background:** Protein intake in relation to resistance training (RT) is often studied under fasted conditions, although most real-world training is performed in a non-fasted state. This study examined whether intra-workout carbohydrate plus protein (CHO:P) co-ingestion enhances muscle hypertrophy compared with isoenergetic carbohydrate (CHO) alone when RT is performed in a non-fasted state. Additionally, baseline testosterone, insulin-like growth factor 1 (IGF-1), capillary density, and maximal oxygen uptake (VO_2_max) were evaluated in relation to gains in muscle fibre cross-sectional area (fCSA). **Methods**: Seventeen physically active young men completed the study (CHO:P, n = 8; CHO, n = 9) as part of an 8-week supervised RT program. Assessments included body composition, physical performance, blood variables, and muscle biopsies obtained before and after the intervention. Fibre-type-specific and distributional (percentile-based) analyses of fCSA were performed. Given the limited sample size, analyses should be considered exploratory. **Results**: No significant between-group differences were observed for any outcome (all *p* > 0.05). In contrast, RT induced significant increases in lean mass, muscle strength, VO_2_max, fat oxidation capacity, and fCSA (all *p* < 0.05). For example, type IIA fCSA median increased from 5232 μm^2^ (P10–P90: 2917–7286) to 5925 μm^2^ (3794–8422 μm^2^). Distributional analysis revealed that type IIA fibre fCSA increased across the full percentile range, indicating a broad hypertrophic response, whereas type I fibres showed more modest and less uniform changes. A marked fibre-type transition was observed, with type IIX fibres decreasing from ~1.2% (0.5–3.2%) to ~0.0% (0.0–1.3%) and IIA/IIX hybrid fibres from ~8.9% (7.3–12.2%) to ~4.4% (1.1–5.6%). Baseline IGF-1 showed an exploratory association with increases in fCSA (*p* = 0.04), whereas testosterone, capillary density, and VO_2_max did not (all *p* > 0.05). **Conclusions**: Resistance training performed in a non-fasted state resulted in robust improvements in muscle hypertrophy, metabolic capacity, and physical performance. No additional benefit of intra-workout protein co-ingestion was detected compared with isoenergetic carbohydrate intake under the present experimental conditions. However, given the small sample size and variability in responses, modest effects cannot be excluded. The observed association between baseline IGF-1 and hypertrophy should be interpreted cautiously and requires confirmation in larger studies.

## 1. Introduction

Skeletal muscle mass and its metabolic capacity are key determinants of mobility, strength, endurance, and metabolic health, including glucose and lipid metabolism. Skeletal muscle exhibits substantial morphological, metabolic, and contractile plasticity in response to altered mechanical loading, with resistance training (RT) representing a primary stimulus for these adaptations [1,2]. Muscle hypertrophy, characterized by an increase in myofibrillar and sarcoplasmic content within individual muscle fibres, is a well-established response to RT [3]. However, the magnitude of hypertrophic adaptation varies considerably between individuals, even when training stimuli are standardized [3,4,5]. The mechanisms underlying this inter-individual variability are not fully understood but are likely influenced by a combination of genetic, physiological, and environmental factors [6]. Several physiological factors have been proposed to contribute to variability in hypertrophic responses. Anabolic hormones such as insulin-like growth factor 1 (IGF-1) and testosterone may influence muscle growth through activation of key intracellular signalling pathways regulating protein synthesis, including the PI3K–Akt–mTOR pathway [7,8]. In addition, skeletal muscle capillarization may affect hypertrophy by influencing oxygen and nutrient delivery, as well as the removal of metabolic by-products [9,10]. Aerobic capacity, often reflected by maximal oxygen uptake (VO_2_max), may further contribute by influencing metabolic efficiency and recovery capacity during repeated training sessions. Together, these factors may interact to modulate the magnitude of muscle hypertrophy in response to RT.

In addition to a well-executed RT routine that promotes muscle hypertrophy [11,12], nutritional strategies play a central role in modulating RT-induced adaptations. Protein ingestion stimulates muscle protein synthesis (MPS), and when combined with RT, repeated elevations in MPS exceeding muscle protein breakdown (MPB) lead to net protein accretion and muscle hypertrophy [13,14,15]. However, while acute increases in MPS are often used to assess anabolic responses, these short-term responses do not necessarily translate directly into long-term hypertrophic adaptations. Moreover, studies investigating protein supplementation during RT have reported inconsistent findings, with some demonstrating greater increases in muscle fibre cross-sectional area (fCSA) compared with isoenergetic carbohydrate provision [16,17,18], whereas others report no additional effect [19,20]. These discrepancies may reflect differences in total daily protein intake, energy balance, carbohydrate availability, and the timing of nutrient ingestion.

Importantly, the effects of protein intake depend not only on total daily intake but also on the timing of ingestion relative to exercise. Many previous studies examining protein ingestion during RT have been conducted in a fasted state [16,17,18], which may exaggerate the anabolic response to protein intake. In the fed state, circulating amino acids and insulin are already elevated, promoting anabolic signalling and suppressing MPB, thereby supporting a positive net protein balance. In contrast, during fasted conditions, reduced amino acid availability and lower insulin concentrations may limit anabolic signalling and increase the relative contribution of protein breakdown. In this context, intra-workout protein ingestion may theoretically influence protein turnover by sustaining amino acid availability and modulating hormonal responses, including insulin, which can contribute to the suppression of MPB. However, when RT is performed in a non-fasted state with adequate nutrient intake before and after exercise, the incremental benefit of additional protein consumed during exercise is likely to be modest. Consequently, it remains unclear whether intra-workout protein provision provides additional benefits beyond those achieved through sufficient total protein intake and nutrient timing around exercise.

In the present study, the non-fasted state refers to participants consuming a meal approximately 1.5–2 h prior to exercise, ensuring the availability of circulating nutrients during training. Importantly, even under controlled training conditions, substantial inter-individual variability in hypertrophic responses to resistance training has been consistently reported. To better capture this variability, muscle fibre-type-specific adaptations were considered. Human skeletal muscle comprises fibre types with distinct contractile and metabolic characteristics, where type I fibres are more oxidative and fatigue-resistant, whereas type II fibres (IIA and IIX) exhibit greater glycolytic capacity and hypertrophic potential, with type I and IIA fibres predominating in the vastus lateralis.

However, it remains unclear whether intra-workout nutrient provision modifies fibre-type-specific hypertrophic adaptations under these conditions or contributes to the observed inter-individual variability in response to resistance training.

To address these gaps, the present randomized, double-blind study investigated the effects of nutrient ingestion during RT in a non-fasted state in physically active young men. Specifically, we aimed to (1) determine whether intra-workout co-ingestion of carbohydrate and protein enhances muscle hypertrophy compared with isoenergetic carbohydrate alone over 8 weeks, and (2) examine whether baseline levels of anabolic hormones, capillarization, and aerobic capacity are associated with gains in muscle fibre cross-sectional area. To complement mean changes, we also performed fibre-type-specific and distributional (percentile-based) analyses of fCSA to characterize heterogeneity in hypertrophic adaptations.

Accordingly, the primary hypothesis was that intra-workout carbohydrate–protein co-ingestion during resistance training would result in greater increases in muscle fibre cross-sectional area compared with isoenergetic carbohydrate alone. Secondary exploratory hypotheses were that baseline levels of IGF-1, testosterone, capillarization, and VO_2_max would be associated with the magnitude of hypertrophic adaptations to resistance training.

## 2. Method

### 2.1. Study Design and Ethical Approval

This study employed a randomized, double-blind, parallel-group design to compare the effects of carbohydrate–protein co-ingestion versus carbohydrate alone during an 8-week resistance training intervention.

To address the study aims, a range of physiological and morphological variables were assessed. Muscle fibre cross-sectional area (fCSA) and capillarization were measured to evaluate hypertrophic and structural adaptations to resistance training and to determine whether carbohydrate–protein co-ingestion enhances muscle growth compared with carbohydrate alone. Whole-body and performance-related variables, including body composition, muscle strength, VO_2_max, and fat oxidation capacity, were assessed to characterize functional and metabolic adaptations accompanying hypertrophy of fCSA. In addition, circulating anabolic hormones (e.g., IGF-1 and testosterone), as well as baseline capillary density and VO_2_max, were evaluated as potential predictors of inter-individual variability in training-induced changes in muscle fCSA.

The study was conducted in accordance with the Declaration of Helsinki and was approved by the Regional Ethical Review Board in Umeå, Sweden (Dnr 09-154M). All participants provided written informed consent prior to participation.

### 2.2. Participants

Physically active young men with limited to moderate resistance training experience were recruited via advertisements at Umeå University, Sweden. Participants were considered recreationally trained, with prior but non-systematic resistance training experience and no history of structured or advanced resistance training programs during the past year. Inclusion criteria were male sex, age 20–35 years, engagement in regular physical activity, and the ability to safely perform heavy resistance training. Exclusion criteria included smoking, lactose intolerance, metabolic or cardiovascular disease, use of medications affecting metabolism or hormonal status, and inability to safely perform heavy resistance training. Twenty-nine individuals met the eligibility criteria. Of these, 17 participants completed the 8-week intervention, all physical testing, blood sampling, and muscle biopsies both before and after the intervention. Reasons for dropout included training interruptions due to prolonged upper respiratory tract infections, unwillingness to undergo post-intervention muscle biopsy, and personal reasons (See flow-chart Supplementary Figure S1). The mean age of completers was 25.5 years (range: 19.4–30.8 years), with mean ages of 25.6 years in the CHO group and 25.2 years in the CHO:P group. All participants completed a standardized medical history questionnaire confirming good health and no contraindications to resistance training.

### 2.3. Randomization and Blinding

Participants were randomly assigned to one of two intervention groups using concealed allocation. Randomization was performed by having each participant draw a sealed envelope containing a slip labelled “1” or “2”, corresponding to the CHO or CHO:P group.

The study was conducted in a double-blind manner. Intervention groups were coded as Group 1 and Group 2, and group allocation remained blinded during data collection, compilation, and statistical analysis until all analyses were completed. Supplement beverages were prepared by a laboratory assistant not involved in data collection or analysis and were standardized in appearance and vanilla flavour, minimizing the risk of participants identifying the intervention. Investigators responsible for outcome assessment and data analysis were blinded to group allocation.

### 2.4. Resistance Training Intervention

Both groups completed the same 8-week supervised resistance training program designed to promote muscle hypertrophy and align with established guidelines for recreationally trained individuals. The program consisted of whole-body exercises incorporating both compound and isolation movements involving concentric and eccentric muscle actions, including leg press, leg extension, leg curl, lat pulldown, shoulder lateral raise, triceps pushdown, chest press, and abdominal crunches. Exercises were performed for 2–4 sets of 8–15 repetitions, with 2–3 min rest between sets. Movement tempo was standardized to approximately 2 s for both concentric and eccentric phases. The program followed a progressive, undulating structure over 8 weeks, with participants completing 2–4 sessions per week. During even-numbered weeks (2, 4, 6, and 8), participants performed the final set of each exercise to concentric failure, whereas during odd-numbered weeks they were instructed to avoid failure training. All sessions were supervised by experienced instructors to ensure proper technique, adherence to the program, and compliance with supplement intake. Each session began with a 10 min cycling warm-up at approximately 75 W. Training was conducted at the Section of Sports Medicine, Umeå University, and the IKSU Sport Centre in Umeå, Sweden. Over the intervention, participants completed an average of 25.6 sessions (CHO: 26.3; CHO:P: 24.8).

### 2.5. Supplementation Intervention and Dietary Control

During each training session, participants consumed a supplement beverage in an amount proportional to body mass. The CHO:P group consumed a beverage providing 0.17 g protein and 0.50 g carbohydrate per kg body mass, while the CHO group consumed an isoenergetic carbohydrate-only beverage providing 0.67 g carbohydrate per kg body mass. Both beverages were standardized to a concentration of 8 g carbohydrate or carbohydrate–protein per 100 mL, divided into four equal portions, and consumed every 15 min throughout the training session. The protein dose was selected to provide a moderate amount of protein during exercise (~12–15 g), which could be ingested without gastrointestinal discomfort. Furthermore, this dose was implemented within a broader nutritional context that included a pre-exercise meal and a standardized post-exercise recovery beverage, ensuring that total daily protein intake aligned with current recommendations for individuals performing resistance training. Participants were instructed to consume a light meal 1.5–2 h before training and a regular meal within 2 h after exercise. To standardize early recovery nutrition, all participants received a post-exercise beverage 25 min after training, providing 0.25 g milk protein and 0.50 g carbohydrate per kg body mass. Additional nutritional or performance-enhancing supplements were prohibited during the study.

Dietary macronutrient intake and energy expenditure were assessed during the week prior to the intervention and during the final week of the RT period. Participants completed a 4-day weighed food record and wore a SenseWear Armband (SWA; BodyMedia Inc., Pittsburgh, PA, USA). The SWA was worn on the upper arm over the triceps (except during water-based activities) and estimated energy expenditure using integrated sensors, including an accelerometer, heat flux sensor, galvanic skin response sensor, skin temperature sensor, and ambient temperature sensor. Data were downloaded and analysed using SenseWear InnerView Research Software 6.1. The SWA has been previously reviewed [21] and validated in healthy adults [22], and considered a reliable and practical tool for comparing energy expenditure between groups in relation to estimated macronutrient intake (Supplementary Table S1).

### 2.6. Physical Performance Testing, Body Composition Assessment, and Blood Analyses

Pre-training assessments were conducted 2–3 days before the first muscle biopsy, and post-intervention assessments were performed two days after the final training session. Body composition was measured using dual-energy X-ray absorptiometry (DXA; Lunar iDXA, GE Healthcare, Waukesha, WI, USA). Body mass, height, lean mass index (LMI), and fat mass index (FMI) were also calculated. Upper-body strength was assessed using a three-repetition maximum (3RM) bench press test. Lower-body strength was assessed as maximal isokinetic knee extensor and flexor torque using a Biodex System 3 Pro dynamometer (Biodex Medical Systems, Inc., New York, NY, USA) at 60°·s^−1^ through a 0–100° range of motion. Venous blood samples were collected in the morning after an overnight fast. Serum was stored at −80 °C until analysis. Testosterone, sex hormone-binding globulin (SHBG), dehydroepiandrosterone sulfate (DHEA-S), and insulin-like growth factor-1 (IGF-1) were analysed using accredited clinical laboratory methods. Maximal oxygen uptake (VO_2_max) and carbon dioxide production were measured during an incremental cycling test to exhaustion using a calibrated ergometer (Ergomedic 839E, Monark, Vansbro, Sweden) and an Oxycon Pro gas analysis system (Jaeger, Würzburg, Germany). Fat oxidation was estimated using the stoichiometric equation described by Frayn, and the area under the curve was calculated from measurements obtained at 120, 160, and 200 W. Data from the present cohort have previously been reported in part in relation to gene expression responses to the resistance training intervention [23]. In that study, changes in whole-body and metabolic variables (e.g., lean mass, VO_2_max, and fat oxidation capacity) were presented descriptively without between-group comparisons and without assessment of muscle fibre cross-sectional area (fCSA). In the present study, these variables are included to characterize the cohort and are analysed in the context of the randomized intervention design, including between-group comparisons and use as covariates. The primary outcomes, including fibre-type specific hypertrophy and capillarization assessed by muscle biopsies, have not been previously reported.

### 2.7. Muscle Biopsy Sampling and Immunohistochemistry

**Figure 1:**
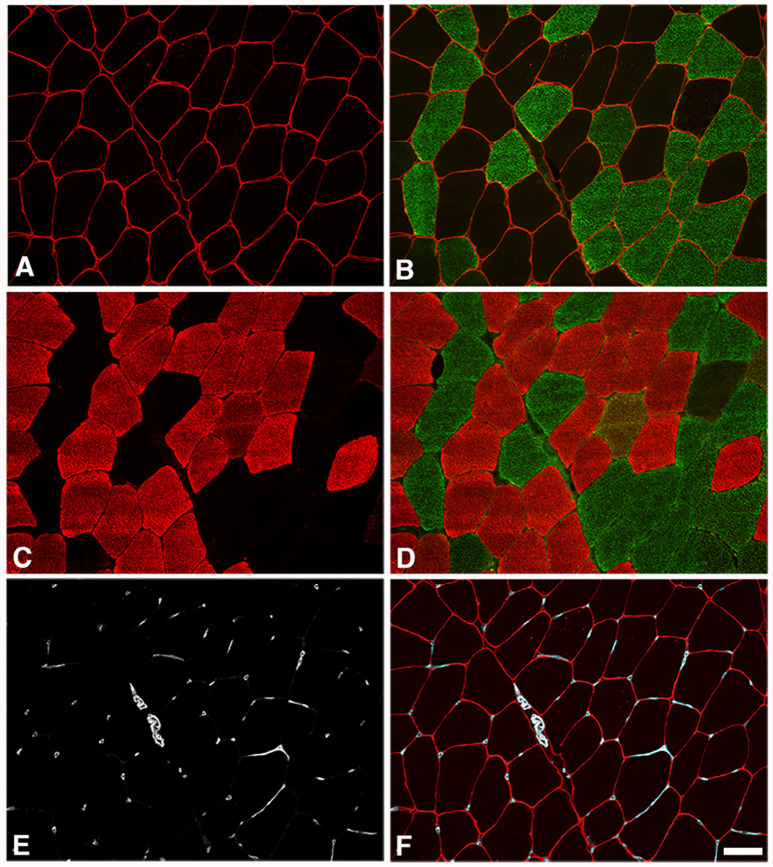
Immunostaining. Serial cross-sections from a control muscle (pre-training) biopsy stained with antibodies directed against different myosin heavy chain (MyHC) and laminin isoforms. (**A**) immunostaining of the basement membrane of muscle fibers using a mAb against laminin α2; (**B**) merged stainings for laminin α2 (red) and MyHC I (green); (**C**) immunostaining for MyHC IIA (red); (**D**) merged staining for MyHC IIA (red) and MyHC I (green); (**E**) immunostaining for capillaries with an antibody against laminin α5 (white); (**F**) merged staining for antibodies directed against α2 (red) and laminin α5 (white). Muscle fibres expressing MyHC I (green), MyHC I + IIA (orange), MyHC IIA (red), MyHC IIA + IIX (weak green), and MyHC IIX (unstained) are marked. Scale bar 50 µm.

Muscle biopsies were obtained from the vastus lateralis under local anaesthesia using the Weil–Blakesley Conchotome technique [24]. Immediately after sampling, visible fat, connective tissue, and blood were removed under a dissection microscope. Pre- and post-intervention samples were collected from opposite legs to avoid repeated sampling from the same site. For each biopsy, two transverse sections mounted on separate slides were analysed. One section was stained using a multicolour approach including antibodies against laminin α2, myosin heavy chain isoforms MyHC I, and MyHC IIA to identify fibre types and determine fibre cross-sectional area. A second section was stained for cell membranes (laminin α2) and capillaries (laminin α5) to assess capillarity. This approach allowed optimal visualization of fibre morphology and capillary structures using staining protocols tailored to each outcome. An example of immunostaining of serial cross-sections is shown in Figure 1. All antibodies used and their dilutions are presented in Table 1. Capillary density was calculated as the number of capillaries per mm^2^ of muscle cross-sectional area. Capillaries around fibres (CAF) were defined as all capillaries located within 5 µm of an individual muscle fibre, and capillaries relative to fibre area (CAFA) were calculated as CAF divided by fibre cross-sectional area × 10^3^.

### 2.8. Morphometric Analysis

Measurements of muscle fibre cross-sectional area (fCSA), fibre type composition, and capillarization were performed using light microscopy and computerized image analysis (Leica DM6000B (Leica Microsystems GmbH, Weitzlar, Germany) with QWin Plus software V3). Three to four randomly selected areas from each biopsy were analysed, and all fibres and capillaries within these regions were manually traced. Fibres were classified according to myosin heavy chain (MyHC) staining patterns as type I, type IIA, type IIX, type I/IIA, or type IIA/IIX. On average, approximately 210 fibres were analysed pre-training and 204 fibres post-training per biopsy. To ensure data quality, all muscle fibre measurements were visually inspected during analysis, and fibre borders were carefully verified against the original immunohistochemical images. Particular attention was given to fibres with unusually large cross-sectional areas to exclude potential artefacts related to staining, sectioning, or image segmentation. No systematic measurement artefacts were identified. To better characterize the distribution of muscle fibre size, analyses were performed at the 10th, 50th (median), and 90th percentiles of fCSA. This approach extends traditional mean-based analyses by capturing changes across the full spectrum of fibre sizes, including smaller and larger fibres that may exhibit distinct adaptive responses to resistance training. By quantifying distributional shifts, this method provides insight into the heterogeneity of hypertrophic adaptations within and between individuals.

### 2.9. Statistical Analysis

Statistical analyses were conducted to evaluate the effects of resistance training (time effect), the nutritional intervention (between-group effect), and associations between baseline variables and hypertrophic adaptations. The primary outcome was mean muscle fibre cross-sectional area (fCSA) across all fibres. Primary analyses focused on changes in fCSA over time and differences between intervention groups. The main effect of time (pre- vs. post-intervention), representing the effect of resistance training, was assessed using linear mixed-effects models with time as a fixed effect and participant as a random effect to account for within-subject repeated measures. This approach allows inclusion of all available data and accounts for inter-individual variability in baseline values and training responses.

Between-group differences (intervention effects, Table 2) were evaluated using analysis of covariance (ANCOVA), with post-intervention values as dependent variables, group as a fixed factor, and the corresponding baseline value included as a covariate. This approach adjusts for baseline differences and provides an unbiased estimate of the intervention effect. Within-group changes over time were additionally explored using the Wilcoxon signed-rank test on main effect of time and are presented as supportive analyses.

Changes in muscle fibre size and capillarization across the distribution (Table 3a,b) were further examined using linear quantile mixed models (LQMM) at the 10th, 50th (median), and 90th percentiles of fCSA, capillaries around fibres (CAF), and capillaries relative to fibre area (CAFA). These models included time (Pre vs. Post) as a fixed effect and participant as a random effect, and interaction terms were used to assess potential intervention effects. The LQMMs were fit independently for each percentile using derivative-free optimization. The confidence intervals were estimated using block bootstrap and checked to be symmetric. Estimated effects and corresponding confidence intervals were derived for each percentile.

Exploratory analyses were conducted to assess associations between post-training fCSA and baseline variables, including physical fitness, body composition, capillary density, and hormone concentrations. Linear regression models were applied with post-training fCSA as the dependent variable and corresponding baseline fCSA included as a covariate.

All statistical analyses were performed using R version 4.5.1 (R Foundation for Statistical Computing, Vienna, Austria), including the lqmm package for quantile mixed modelling. Statistical significance was set at *p* < 0.05. No adjustment for multiple comparisons was applied. The primary outcome was interpreted as confirmatory, whereas all additional analyses, including fibre-type-specific outcomes, distributional analyses, and regression models, were considered exploratory and interpreted with caution. No a priori sample size calculation was performed for the present study. The sample size was primarily determined by practical considerations, including the complexity of the intervention and the use of invasive muscle biopsy procedures. Accordingly, the study should be considered exploratory in nature and was not formally powered to detect small or moderate between-group differences. The ability to detect modest intervention effects may therefore be limited.

## 3. Results

### 3.1. Physical Fitness, Hormonal Status, and Body Composition

Results for physical performance, body composition, and hormonal variables are presented in Table 2. No statistically significant between-group differences were observed at post-intervention (all *p* > 0.05, ANCOVA adjusted for baseline), indicating that intra-workout protein co-ingestion did not influence these outcomes.

In contrast, analyses of the main effect of time revealed significant (*p* < 0.05) training-induced adaptations (Table 2), based on LQMMs as described in the Methods. Specifically, increases were observed in total body mass, whole-body lean mass, and regional lean mass in both arms and legs. In addition, participants demonstrated improvements in maximal oxygen uptake (VO_2_max), fat oxidation capacity, and muscle strength, as assessed by the 3RM bench press and isokinetic leg extension tests.

No statistically significant differences were observed between groups in energy intake, macronutrient composition, or estimated energy expenditure at baseline or post-intervention (all *p* > 0.05; Supplementary Table S1). On training days, total protein intake was within recommended ranges in both groups and did not differ significantly between groups, although the CHO group tended to exhibit slightly higher total energy and protein intake.

In addition to their habitual dietary protein intake, participants in the CHO:P group consumed supplemental protein and carbohydrates both during and after each workout session. In contrast, the CHO group consumed carbohydrates only during exercise, followed by protein and carbohydrates post-exercise. On average, participants in the CHO:P group ingested 32.9 g of protein per workout (combined intake during and after exercise), whereas those in the CHO group consumed 19.4 g over the same period. This supplementation resulted in a median total protein intake of 1.65 g·kg^−1^ body mass (IQR: 0.46) in the CHO:P group and 1.81 g·kg^−1^ (IQR: 0.40) in the CHO group on training days. These values reflect total protein intake from both dietary sources and supplementation, based on data collected during week 8 of the intervention. No statistically significant between-group differences were observed (*p* > 0.05). However, the CHO group tended to exhibit higher total energy expenditure, as well as greater overall energy and protein intake, compared with the CHO:P group. Detailed data on dietary energy intake, macronutrient composition, and energy expenditure are presented in Supplementary Table S1.

### 3.2. Muscle Fibre Size, Capillarization, and Fibre-Type Distribution

Between-group comparisons are presented in Table 3a. No statistically significant differences were observed between supplementation groups for muscle fibre cross-sectional area (fCSA) or capillarization at any percentile (P10–P90), indicating that protein co-ingestion during exercise did not influence these outcomes.

In contrast, main effect of time revealed significant training-induced adaptations (Table 3b). Type IIA fCSA increased significantly across the distribution (P10, P50, and P90), while fCSA main effect of time across all fibre types also increased. For type I fibres, a significant increase was observed at the median (P50) level only.

Capillarization showed limited changes following training. However, capillaries relative to fibre area (CAFA) decreased significantly in type IIA fibres at the median and upper percentiles, indicating that fibre hypertrophy exceeded changes in capillary supply. No significant changes were observed in capillaries around fibres (CAF) or CAFA in type I fibres. Considerable inter-individual variability in hypertrophic responses was observed. For type I fibres, the median fCSA increased in nine participants and decreased in three, while for type IIA fibres, increases were observed in the majority of participants, with decreases in a small number of individuals. In addition, a small number of fibres with exceptionally large cross-sectional areas were observed in some participants, indicating substantial heterogeneity in fibre size distribution. Visual inspection of the original muscle cross-sections confirmed that these large fibres were not attributable to measurement artefacts or segmentation errors, but rather reflected true biological variability. Importantly, the use of percentile-based analyses reduces the influence of such extreme values on the overall interpretation. For example, in one participant’s pre-training biopsy, several type IIA fibres ranged from 13,852 to 17,267 µm^2^, while another participant exhibited unusually large type I fibres ranging from 12,054 to 16,744 µm^2^, with percentile values remaining within typical ranges in both cases.

Beyond these fibre size adaptations, resistance training also induced marked shifts in fibre-type composition. The proportion of type IIX fibres decreased from 1.2% (IQR: 0.5–3.2%) at baseline to 0.0% (IQR: 0.0–1.3%) post-training, while hybrid type IIA/IIX fibres were reduced from 8.9% (IQR: 7.3–12.2%) to 4.4% (IQR: 1.1–5.6%). These changes indicate a transition toward a more oxidative fibre-type profile (Supplementary Figure S2). No statistically significant between-group differences were observed for any fCSA or capillarization outcomes. The wide confidence intervals for between-group differences indicate substantial variability and suggest that both positive and negative effects cannot be excluded.

Correlation analyses revealed that the relative change in type I fibre fCSA was negatively associated with baseline fCSA (r = −0.65, *p* = 0.01), whereas the corresponding association for type IIA fibres was positive but did not reach statistical significance (r = 0.47, *p* = 0.06). No statistically significant between-group differences were observed for fCSA or capillarization across fibre types (Table 3a). The wide confidence intervals for between-group differences indicate substantial variability and suggest that both positive and negative effects cannot be excluded. Despite the absence of between-group differences, a significant decrease in CAFA was observed in type IIA fibres following training (Table 3b), indicating that fibre hypertrophy exceeded changes in capillarization in this fibre type.

### 3.3. Associations with Muscle Fibre Hypertrophy

Exploratory analyses examining associations between baseline variables and post-training fCSA are presented in Table 4. Baseline IGF-1 levels showed a positive exploratory association with type IIA fCSA following the intervention (*p* = 0.04), whereas no significant associations were observed for testosterone, capillarization, or VO_2_max.

## 4. Discussion

The present randomized, placebo-controlled study examined whether protein co-ingestion during resistance exercise enhances adaptations in muscle fibre size, capillarization, and related physiological outcomes. Protein co-ingestion during exercise did not result in additional detectable benefits compared with carbohydrate alone. However, the relatively small sample size (n = 17) limits statistical power, and the wide confidence intervals observed for between-group comparisons indicate substantial variability, suggesting that both positive and negative effects of the intervention cannot be excluded. These findings should therefore be interpreted with caution.

Importantly, the present study examined the effect of additional protein intake during exercise under conditions where participants were advised to consume a light meal prior to each resistance training session, received protein supplementation post-exercise, and achieved relatively high total daily protein intakes. Moreover, total protein intake was numerically higher in the CHO group compared with the CHO:P group; however, this likely reflects the concurrently higher estimated energy expenditure and total energy intake observed in this group, rather than a systematic difference in dietary composition. These factors should be considered when interpreting the findings, as they may have limited the potential for additional intra-workout protein to further enhance training adaptations.

Notably, many studies examining the effects of protein supplementation during resistance training are conducted under fasted conditions, which may promote a more catabolic environment and may not reflect typical resistance training conditions aimed at optimizing hypertrophy. In contrast, participants in the present study were not fasted and achieved relatively high total protein intakes. Under such conditions, the lack of additional benefit from intra-exercise protein ingestion may reflect an already favourable nutritional environment for muscle adaptation, in which the potential for further enhancement appears to be attenuated.

Resistance training induced robust increases in muscle fibre cross-sectional area (fCSA), lean mass, muscle strength, fat oxidation capacity, and VO_2_max; however, no statistically significant differences were observed between the CHO:P and CHO groups. These findings suggest that protein co-ingestion during exercise did not result in additional detectable benefits beyond carbohydrate alone under the conditions of the present study. The absence of additional hypertrophic effects with protein co-ingestion during exercise may be explained by several interacting factors.

First, all participants performed RT in a non-fasted state, consuming a meal 1.5–2 h prior to exercise and a standardized protein–carbohydrate beverage during early recovery. Under such conditions, amino acids stimulate muscle protein synthesis primarily through activation of mTORC1 signalling pathways, whereas insulin plays a permissive and predominantly anti-catabolic role by suppressing protein breakdown and facilitating net protein accretion [25,26,27]. Together, these signals act synergistically, with amino acids providing the primary anabolic stimulus and insulin promoting a favourable anabolic milieu by limiting proteolysis and supporting nutrient-dependent signalling. In this context, carbohydrate intake during exercise helps maintain or elevate circulating insulin levels, thereby attenuating muscle protein breakdown and promoting a more favourable net protein balance. Consequently, anabolic signalling pathways may operate near maximal capacity under these conditions, limiting the potential for additional stimulation from intra-exercise protein intake.

Second, total daily protein intake in both groups was within or above recommended levels (~1.6–1.8 g·kg^−1^·day^−1^) [28], suggesting the presence of a ceiling effect. Previous meta-analyses indicate that protein intakes above ~1.6 g·kg^−1^·day^−1^ do not confer further increases in fat-free mass during RT [29]. Although not statistically different, the CHO group tended to exhibit slightly higher energy and protein intake, which may have attenuated potential differences between supplementation strategies. Thus, the efficacy of protein supplementation appears to depend on the overall nutritional context. Accordingly, the absence of additional benefits from intra-exercise protein ingestion should be interpreted within a context of already adequate total protein intake, rather than as evidence for an absence of protein effects per se.

These findings contrast with studies reporting greater hypertrophy with protein supplementation [16,17,18]. Importantly, many of these studies were conducted under fasted conditions, where reduced insulin and amino acid availability limit anabolic signalling and increase proteolysis [30,31,32], potentially enhancing responsiveness to protein intake [33,34,35]. In applied settings, however, RT is typically performed in a fed state [28,36], which may explain discrepancies between experimental and real-world conditions.

At the muscle fibre level, RT induced marked hypertrophic adaptations, particularly in type IIA fibres, which increased across the fibre size distribution, whereas type I fibres showed more modest changes. Importantly, the percentile-based analytical approach allowed identification of fibre-specific adaptations across the size distribution, highlighting the heterogeneous nature of muscle hypertrophy. By capturing changes in both smaller and larger fibres, this approach extends traditional mean-based analyses and provides insight into distributional shifts that may otherwise remain undetected. Accordingly, this method offers a more nuanced understanding of inter-individual variability in hypertrophic responses.

Substantial inter-individual variability in hypertrophic responses was observed. This variability was evident both between participants and within individuals, including the presence of exceptionally large fibres despite typical percentile values. These findings align with classical observations of variability in fibre size within motor units [37] and likely reflect differences in local mechanical loading, metabolic support, and cellular adaptations.

Resistance training also induced marked shifts in fibre-type composition, with reductions in type IIX and hybrid IIA/IIX fibres and a transition toward more oxidative phenotypes. These fibre-type transitions are primarily driven by changes in myosin heavy chain isoform expression within individual fibres, such that fibres can shift from type IIX to IIA phenotypes through coordinated downregulation and upregulation of specific isoforms, rather than through fibre loss and replacement [38,39]. Collectively, these changes reflect coordinated adaptations in both contractile and metabolic properties.

Interestingly, hypertrophic adaptations were not accompanied by increased capillarization. Instead, capillary density relative to fibre area (CAFA) decreased in type IIA fibres, most likely reflecting increases in muscle fibre cross-sectional area exceeding the rate of capillary proliferation. This pattern is consistent with previous observations following resistance training and does not necessarily indicate a loss of capillaries, but rather a proportional lag in angiogenesis relative to fibre growth. These findings highlight that hypertrophic and vascular adaptations may occur on different time scales and underscore the complexity of skeletal muscle adaptation. This interpretation is further supported by the absence of changes in capillaries around fibres (CAF), suggesting that capillary number was largely preserved.

Regarding systemic predictors, baseline IGF-1 levels were positively associated with increases in type IIA fCSA, whereas no such association was observed for testosterone. However, this finding should be interpreted with caution, as the analysis was exploratory and conducted in a relatively small sample. Previous studies have reported inconsistent associations between IGF-1 and hypertrophy [40,41,42], and the present findings should therefore be considered hypothesis-generating.

In addition to structural adaptations, RT induced significant improvements in VO_2_max and fat oxidation capacity. Although carbohydrate availability is known to influence intracellular signalling pathways regulating metabolic adaptations [43], and insulin is well established to attenuate lipid oxidation and promote carbohydrate utilisation [44], the present findings demonstrate that resistance training induced significant improvements in fat oxidation capacity despite carbohydrate ingestion during exercise. This suggests that chronic training-induced adaptations may override or compensate for acute substrate-mediated effects, allowing for concurrent improvements in both anabolic and oxidative metabolism. In addition, the observed increase in VO_2_max may be partly explained by cardiovascular adaptations to resistance training. Although endurance exercise is typically considered the primary stimulus for improving aerobic capacity, resistance training has been shown to increase VO_2_max, particularly in individuals with lower baseline aerobic fitness [45]. Notably, hypertrophy-oriented resistance training is associated with substantial acute increases in systolic blood pressure during exercise, in some cases exceeding 300–400 mmHg during multi-joint exercises [46], which may provide a stimulus for cardiovascular adaptation. Repeated exposure to such hemodynamic loading may induce physiological cardiac remodelling, including increases in myocardial mass and left ventricular wall thickness [47]. These adaptations, together with potential increases in blood volume and cardiac output, may contribute to improved stroke volume and oxygen delivery, thereby supporting increases in VO_2_max [48]. However, these mechanisms remain speculative in the present context. As cardiac structure and function were not assessed in this study, the potential contribution of central cardiovascular adaptations, such as changes in stroke volume or cardiac output, cannot be determined.

From a broader physiological and applied perspective, nutritional strategies play an important role in modulating training adaptations. The present findings support the view that protein supplementation is context-dependent, with limited additional benefit when total protein intake is sufficient and training is performed in a fed state [20,29,49]. From a practical perspective, these findings are particularly relevant for recreationally active individuals, for whom additional protein ingestion during exercise may offer limited incremental benefit.

Finally, dietary intake was assessed using weighed food records, which are subject to known limitations, including misreporting and changes in intake during the recording period [50,51]. However, as the same methodology was applied across both groups, any resulting bias is likely to be non-differential.

## 5. Strengths and Limitations

Several limitations should be considered when interpreting the present findings. First, no a priori sample size calculation was performed, and the final sample size was relatively small (n = 17), with attrition from 29 initially enrolled participants. This limited statistical power, particularly for detecting between-group differences, and contributed to uncertainty in the effect estimates, as reflected by wide confidence intervals. Accordingly, the absence of statistically significant between-group differences should be interpreted with caution. In this context, the study should be considered exploratory in nature and was not formally powered to detect modest between-group differences. Second, multiple statistical comparisons were conducted without formal adjustment, and several analyses, including associations between baseline biomarkers (e.g., IGF-1 and testosterone) and hypertrophic outcomes, were exploratory in nature and should be interpreted cautiously. Third, dietary intake was assessed using weighed food records, which may be subject to misreporting, and continuous dietary monitoring throughout the intervention was not performed. In addition, participants were advised to consume a pre-exercise meal, and both groups received protein supplementation post-exercise, resulting in relatively high total daily protein intakes, with numerically higher intake in the CHO group. These factors may have limited the ability to detect additional benefits of intra-exercise protein ingestion. Fourth, the homogeneous study population of physically active young men limits generalizability to other populations.

Despite these limitations, the study has several important strengths. All training sessions were supervised by experienced personnel, ensuring correct exercise execution and individualized progressive overload, which enhances the validity and reproducibility of the training stimulus. Energy balance was carefully assessed through the combined measurement of dietary intake and energy expenditure using wearable monitoring (SenseWear), which strengthens the interpretation of body composition and metabolic outcomes. Body composition was evaluated using segmental DXA, and the observed increases in lean mass were consistent with muscle fibre hypertrophy assessed by biopsy, providing converging evidence across whole-body and tissue-level measurements. Importantly, hypertrophy was assessed at the muscle fibre level, allowing detailed characterization of adaptations. The use of linear quantile mixed models enabled analysis across the fibre size distribution, providing novel insight into heterogeneous responses that are not captured by mean-based approaches. Furthermore, the study assessed a broad range of outcomes, including muscle morphology, capillarization, metabolic variables, and performance measures. Notably, the concurrent observation of increased fat oxidation capacity alongside muscle hypertrophy, despite carbohydrate ingestion during exercise, provides an interesting and physiologically relevant finding that warrants further investigation.

## 6. Conclusions

Under conditions of pre-exercise feeding, post-exercise protein–carbohydrate recovery intake, and adequate total daily protein intake, adding protein to the intra-workout beverage did not produce detectable additional benefits for muscle fibre hypertrophy compared with isocaloric carbohydrate intake. These findings suggest that well-structured resistance training performed under conditions of sufficient energy and protein availability, in line with current sports nutrition guidelines, can effectively promote muscular and metabolic adaptations. This highlights the importance of overall nutritional context rather than the timing of additional intra-exercise protein intake. However, larger studies are needed to determine whether intra-workout protein provides additional benefits under different nutritional or training conditions, particularly when protein intake is suboptimal or training is performed in the fasted state.

In addition, resistance training induced shifts in muscle fibre-type composition toward more oxidative phenotypes and improved fat oxidation capacity despite carbohydrate ingestion, indicating that training adaptations may override acute metabolic effects. Finally, the use of percentile-based analyses further provided novel insight into the distribution of fibre hypertrophy, highlighting heterogeneous adaptations not captured by mean values alone. Overall, these findings support the view that resistance training is a key driver of muscular and metabolic adaptation, while the benefits of protein supplementation appear to depend on the broader nutritional context.

## Figures and Tables

**Table 1:** Antibodies used for immunohistochemical analyses.

Antibody	Specificity	Gene *	Dilution	Source
A4.840	MyHC-1	*MYH7*	1:30	DSHB Cat# A4.840, RRID:AB_528384
A4.74	MyHC-2a	*MYH2*	1:400	DSHB Cat# A4.74, RRID:AB_528383
N2.261	MyHC-1 MyHC-2a	*MYH7* *MYH2*	1:50	DSHB Cat# N2.261, RRID:AB_531790
4C7	Laminin α5 chain	*LAMA5*	1:200	Agilent Cat# M0638, RRID:AB_2249754
5H2.	Laminin α2 chain	*LAMA2*	1:1000	Leica Biosystems Cat# NCL-MEROSIN, RRID:AB_442108

MyHC, myosin heavy chain. Mab(s), monoclonal antibody(ies). Mabs A4.840, A4.74, and N2.261 were obtained from The Developmental Studies Hybridoma Bank, developed under the auspices of the NICHD and maintained by Dept of Biological Sciences, The University of Iowa, Iowa City, IA, USA. * Official gene nomenclature according to OMIM. (http://www.ncbi.nlm.nih.gov/omim/, accessed on 1 July 2026).

**Table 2:** Participant characteristics, physical performance, metabolic, and hormonal variables before and after 8 weeks of resistance training.

	CHO, n = 9		CHO:P, n = 8		All, n = 17, Time-Effect		
Variable	Pre	Post	Pre	Post	Pre	Post	*p*
**Aerobic fitness & strength**							
VO_2_max (L·min^−1^)	4.2 (3.4, 5.2)	4.7 (3.9, 6.0)	4.0 (3.1, 5.1)	4.7 (4.1, 5.8)	4.1 (3.1, 5.2)	4.7 (3.9, 6.0)	0.001
Fat oxidation rate (AUC, g)	0.9 (0.4, 1.5)	1.4 (0.5, 2.5)	0.3 (0.1, 1.9)	1.2 (0.8, 2.1)	0.8 (0.1, 1.9)	1.4 (0.5, 2.5)	<0.001
Leg extension peak torque, (Nm)	256 (194, 288)	258 (204, 323)	239 (209, 303)	253 (239, 339)	245 (194, 303)	256 (204, 339)	0.004
3RM bench press (kg)	75.0 (55.0, 105)	83.0 (65.0, 110)	71.5 (60.0, 110)	75.0 (50.0, 110)	75.0 (55.0, 110)	80.0 (50.0, 110)	0.006
**Hormones**							
Total Testosterone (nmol/L)	23.0 (13.0, 31.0)	24.0 (16.0, 35.0)	21.5 (17.0, 33.0)	23.0 (18.0, 29.0)	22.0 (13.0, 33.0)	23.5 (16.0, 35.0)	0.394
SHBG (nmol/L)	29.0 (23.0, 69.0)	32.5 (23.0, 51.0)	33.5 (20.0, 39.0)	34.5 (25.0, 64.0)	31.0 (20.0, 69.0)	34.0 (23.0, 64.0)	0.268
DHEA (ng/L)	7.3 (5.9, 15.8)	7.6 (6.3, 11.7)	8.2 (5.1, 13.7)	7.6 (6.0, 14.4)	8.0 (5.1, 15.8)	7.6 (6.0, 14.4)	0.234
IGF-1 (µg/L)	261 (165, 331)	257 (195, 308)	231 (177, 384)	276 (204, 316)	244 (165, 384)	267 (195, 316)	0.464
**Body weight & composition**							
Body mass (kg)	77.7 (62.5, 90.3)	81.5 (63.1, 94.0)	78.2 (71.5, 91.0)	80.1 (75.1, 94.1)	77.7 (62.5, 91.0)	81.5 (63.1, 94.1)	0.001
Fat (%)	19.7 (12.0, 22.9)	19.2 (12.4, 22.1)	21.6 (15.7, 26.6)	21.1 (14.2, 26.2)	20.8 (12.0, 26.6)	20.0 (12.4, 26.2)	0.093
Fat mass (kg)	14.6 (8.3, 18.9)	14.4 (8.8, 19.1)	16.6 (11.5, 20.1)	16.0 (10.5, 20.7)	15.3 (8.3, 20.1)	15.8 (8.8, 20.7)	0.776
Fat mass index	4.4 (2.7, 5.8)	4.4 (2.9, 5.9)	4.8 (3.2, 6.6)	4.8 (2.9, 6.8)	4.6 (2.7, 6.6)	4.6 (2.9, 6.8)	0.795
Lean mass (kg)	60.9 (49.6, 73.6)	62.2 (51.6, 74.2)	59.8 (52.0, 68.2)	61.3 (54.4, 69.7)	60.9 (49.6, 73.6)	62.2 (51.6, 74.2)	<0.001
Lean mass index	18.7 (16.9, 21.0)	19.7 (17.6, 21.5)	17.8 (16.1, 19.9)	18.7 (16.3, 20.2)	18.2 (16.1, 21.0)	19.0 (16.3, 21.5)	<0.001
Lean mass legs (kg)	21.6 (16.8, 25.0)	23.3 (17.6, 26.5)	21.5 (19.6, 24.3)	22.5 (20.9, 25.5)	21.6 (16.8, 25.0)	23.0 (17.6, 26.5)	<0.001
Lean mass arms (kg)	8.0 (5.9, 9.9)	8.6 (6.3, 10.6)	7.8 (6.5, 10.0)	8.2 (7.2, 10.4)	7.9 (5.9, 10.0)	8.5 (6.3, 10.6)	<0.001

Physical performance, body composition, metabolic, and hormonal variables before and after the intervention. Values are presented as median (50th percentile) with 10th and 90th percentiles (P10–P90). CHO: carbohydrate group (n = 9); CHO:P: carbohydrate plus protein group (n = 8). Pre: baseline; Post: after 8 weeks of resistance training. VO_2_max: maximal oxygen uptake; 3RM: three-repetition maximum; Fat oxidation is expressed as area under the curve (AUC), calculated from 1 min mean values at 120, 160, and 200 W during incremental exercise. Hormone levels refer to circulating concentrations of testosterone and insulin-like growth factor-1 (IGF-1). Body composition variables include body mass, fat-free mass, and fat mass. Percentiles are reported to describe the distribution of responses within each group. *p*-values represent the main effect of time (pre vs. post), assessed using linear mixed-effects models, as described in Section 2.9. No statistically significant between-group differences were observed for any variable at baseline or post-intervention (all *p* > 0.05).

**Table 3:** (**a**) Between-group comparisons of muscle fibre cross-sectional area (fCSA) and capillarization before and after 8 weeks of resistance training in the carbohydrate (CHO, n = 9) and carbohydrate plus protein (CHO:P, n = 8) groups. (**b**) Training-induced changes in muscle fibre cross-sectional area (fCSA) and capillarization in all participants (n = 17).

**(a)**					
**Variable**	**CHO Pre**	**CHO Post**	**CHO:P Pre**	**CHO:P Post**	**Δ, P50 ± CI (*p*)**
**Type I** (µm^2^)	4023 (1738, 6020)	4278 (2052, 6640)	4198 (2448, 6238)	4564 (2585, 6691)	−166 ± 760 (0.66)
**Type IIA** (µm^2^)	5120 (2451, 7377)	6154 (3404, 8552)	5365 (3607, 7120)	5721 (4116, 8262)	−292 ± 828 (0.48)
**All fibres** (µm^2^)	4624 (2161, 6865)	5155 (2734, 8018)	4755 (2934, 6772)	5248 (3254, 7748)	−65 ± 621 (0.83)
**CAF Type I**	4 (2, 7)	4 (2, 7)	4 (2, 7)	4 (2, 7)	0.00 ± 0.93 (1.00)
**CAF Type IIA**	4 (2, 6)	5 (2, 7)	4 (2, 6)	4 (2, 6)	−0.31 ± 1.04 (0.55)
**CAF All fibres**	4 (2, 6)	4 (2, 7)	4 (2, 6)	4 (2, 6)	−0.58 ± 1.31 (0.38)
**CAFA Type I**	1.11 (0.58, 2.23)	1.06 (0.59, 1.92)	1.05 (0.59, 1.62)	0.96 (0.50, 1.56)	0.00 ± 0.20 (1.00)
**CAFA Type IIA**	0.83 (0.38, 1.62)	0.78 (0.37, 1.30)	0.79 (0.39, 1.23)	0.69 (0.27, 1.07)	0.00 ± 0.22 (1.00)
**CAFA All fibres**	0.91 (0.39, 1.79)	0.90 (0.45, 1.60)	0.87 (0.43, 1.44)	0.77 (0.33, 1.31)	−0.06 ± 0.18 (0.53)
(**b**)					
**Variable**	**Pre (Median P10–P90)**	**Post (Median P10–P90)**	**Δ, P50 ± CI (*p*)**	**Δ, P10 ± CI (*p*)**	**Δ, P90 ± CI (*p*)**
**Type I** (µm^2^)	4108 (2095, 6126)	4372 (2352, 6670)			
**Type IIA** (µm^2^)	5232 (2917, 7286)	5925 (3794, 8422)	379 ± 426 (0.08)	379 ± 426 (0.08)	379 ± 426 (0.08)
**All fibres** (µm^2^)	4694 (2424, 6825)	5211 (2955, 7903)			
**CAF Type I**	4 (2, 7)	4 (2, 7)	294 ± 414 (0.16)	294 ± 414 (0.16)	294 ± 414 (0.16)
**CAF Type IIA**	4 (2, 7)	4 (2, 7)			
**CAF All fibres**	4 (2, 6)	4 (2, 7)	272 ± 704 (0.44)	272 ± 704 (0.44)	272 ± 704 (0.44)
**CAFA Type I**	1.07 (0.59, 1.82)	1.02 (0.54, 1.72)			
**CAFA Type IIA**	0.81 (0.38, 1.41)	0.73 (0.32, 1.20)	876 ± 478 (0.00)	876 ± 478 (0.00)	876 ± 478 (0.00)
**CAFA All fibres**	0.89 (0.41, 1.58)	0.84 (0.39, 1.48)			

(a) Values are presented as median with 10th and 90th percentiles (P10–P90). Pre: baseline; Post: after the intervention. Between-group differences (Δ) represent estimated differences in median training-induced changes and were estimated using linear quantile mixed models (LQMM), with post-intervention values as the dependent variable, group as a fixed factor, and corresponding baseline values as covariates. Data are presented as estimate ± 95% confidence interval (CI) (*p*-value), where CIs are symmetric. CAF: capillaries around fibres; CAFA: capillary-to-fibre area ratio. No statistically significant between-group differences were observed (*p* ≥ 0.05). (b) Training-induced changes (Δ, estimate 95% CI (*p*-value)) in muscle fibre cross-sectional area (fCSA) and capillarization in all participants (n = 17). Values are presented as median with 10th and 90th percentiles (P10–P90) at baseline (Pre) and after 8 weeks of resistance training (Post). Estimated changes (Pre to Post) at the median (P50), 10th (P10), and 90th (P90) percentiles were derived using linear mixed-effects models and linear quantile mixed models, with time included as a fixed effect and participant as a random effect. CAF: capillaries around fibres; CAFA: capillary-to-fibre area ratio. No adjustment for multiple comparisons was applied, and these results should be interpreted with caution.

**Table 4:** Associations between baseline variables and post-training fibre cross-sectional area (fCSA).

Variable	Type I β (95% CI)	*p*	Type IIA β (95% CI)	*p*
**Testosterone** (nmol·L^−1^)	34.5 (−48.0, 117.0)	0.38	14.8 (−77.8, 107.4)	0.74
**IGF-1** (µg·L^−1^)	5.6 (−1.5, 12.6)	0.11	7.7 (0.4, 15.2)	0.04
**VO_2_max** (L·min^−1^)	522.6 (−274.7, 1320.0)	0.18	724.2 (−111.6, 1560.0)	0.08
**CAF** (capillaries/fibre)	−390.3 (−961.3, 180.6)	0.16	−311.3 (−938.2, 315.6)	0.30

Exploratory adjusted linear regression analyses were conducted to examine associations between baseline variables and post-training fibre cross-sectional area (fCSA). Regression coefficients (β), 95% confidence intervals (CIs), and *p*-values are presented for each model. All models were adjusted for baseline fCSA. The β coefficient represents the expected change in post-training fCSA per unit increase in the predictor variable. These analyses are exploratory and should be interpreted with caution.

## Data Availability

The investigators do not have permission to disclose the data under the terms of the ethics approval and participant consent.

## Supplementary Materials

The following supporting information can be downloaded at: https://www.mdpi.com/article/10.3390/nu18142307/s1, Table S1. Estimated energy expenditure and dietary intake before and after the intervention; Figure S1. Participant flow diagram. A total of 29 participants were assessed for eligibility and randomized to either the CHO group (n = 14) or the CHO group (n = 15). Five participants in the CHO group and seven in the CHO group were lost to follow-up due to personal reasons or upper respiratory tract infection (URTI). Consequently, 9 participants in the CHO group and 8 participants in the CHO group completed the study and were included in the final analysis; Figure S2. Muscle fibre-type distribution before and after 8 weeks of resistance training. The figure provides a descriptive overview of fibre-type distribution (mean ± SD) before and after 8 weeks of resistance training in all participants (n = 17), illustrating a shift toward a more oxidative profile. Statistical analyses are presented in the main text.

## Abbreviations

AUC	Area Under the Curve
B.W.	Body Weight
CAF	Capillaries Around Fibre
CAFA	Capillaries per Fibre Cross-Sectional Area
CHO	Carbohydrate
CHO:P	Carbohydrate plus Protein
CS	Citrate Synthase
DHEA-S	Dehydroepiandrosterone Sulphate
DXA	Dual-Energy X-Ray Absorptiometry
EE	Energy Expenditure
FFM	Fat-Free Mass
fCSA	Fibre Cross-Sectional Area
FMI	Fat Mass Index
IHC	Immunohistochemistry
IGF-1	Insulin-Like Growth Factor-1
IQR	Interquartile Range
LMI	Lean Mass Index
LQMM	Linear Quantile Mixed Model
MAbs	Monoclonal Antibodies
MPB	Muscle Protein Breakdown
MPS	Muscle Protein Synthesis
MU	Motor Unit
MyHC	Myosin Heavy Chain
OxCaps	Oxidative Capacities (VO_2_max, fat oxidation capacity)
RT	Resistance Training
SHBG	Sex Hormone-Binding Globulin
SWA	SenseWear Armband
VCO_2_	Carbon Dioxide Production
VO_2_max	Maximal Oxygen Uptake
