# Supplementary material for: Effect of Intra-Workout Protein–Carbohydrate Co-Ingestion Versus Isocaloric Carbohydrate During Resistance Training on Muscle Fibre Hypertrophy and Oxidative Capacities in Young Men: A Randomized Controlled Trial
Source: Nutrients. 2026 Jul 14;18(14):2307. doi: 10.3390/nu18142307 (PMC13414563; doi:10.3390/nu18142307)
Supplement: Supplementary file 1 [file nutrients-18-02307-s001.zip › nutrients-4315078-supplementary.pdf]

Table S1. Estimated energy expenditure and dietary intake before and after the intervention

	CHO group, n = 9		CHO:P group, n = 8	
**Variable**	**Pre**	**Post**	**Pre**	**Post**
**Estimated energy expenditure** (kcal·day^{-1})	3095 (603)	3224 (646)	2787 (305)	2838 (167)
**Energy intake** (kcal·day^{-1})	2685 (275)	2996 (323)	2505 (321)	2678 (456)
**Carbohydrate intake** (g·kg^{-1}·day^{-1})	3.84 (0.68)	4.11 (0.34)	3.90 (1.19)	3.89 (0.41)
**Fat intake** (g·kg^{-1}·day^{-1})	1.10 (0.29)	1.44 (0.32)	1.06 (0.38)	1.20 (0.16)
**Protein intake** (g·kg^{-1}·day^{-1})	1.48 (0.63)	1.56 (0.32)	1.14 (0.70)	1.23 (0.29)
**Protein intake** (% of total energy)	20.8 (6.4)	16.4 (3.8)	15.6 (4.4)	16.9 (3.8)

Values are presented as median (interquartile range, IQR). No significant between-group differences were observed in energy or macronutrient intake (all $p > 0.05$, Mann–Whitney U test). Minor within-group changes over time were observed for selected variables. However, these did not differ between groups. Data are presented for descriptive purposes, and key statistical outcomes are reported in the main text.

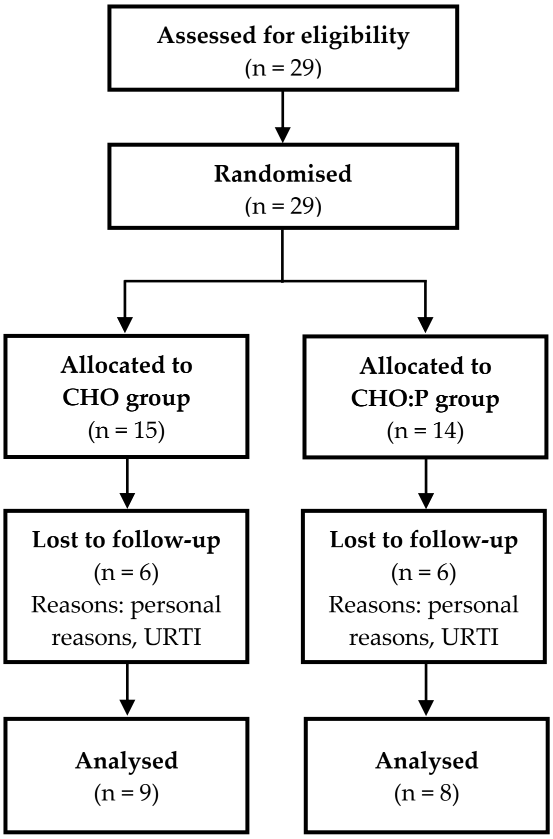

Figure S1. Participant flow diagram. A total of 29 participants were assessed for eligibility and randomized to either the CHO group (n = 14) or the CHO group (n = 15). Five participants in the CHO group and seven in the CHO group were lost to follow-up due to personal reasons or upper respiratory tract infection (URTI). Consequently, 9 participants in the CHO group and 8 participants in the CHO group completed the study and were included in the final analysis.

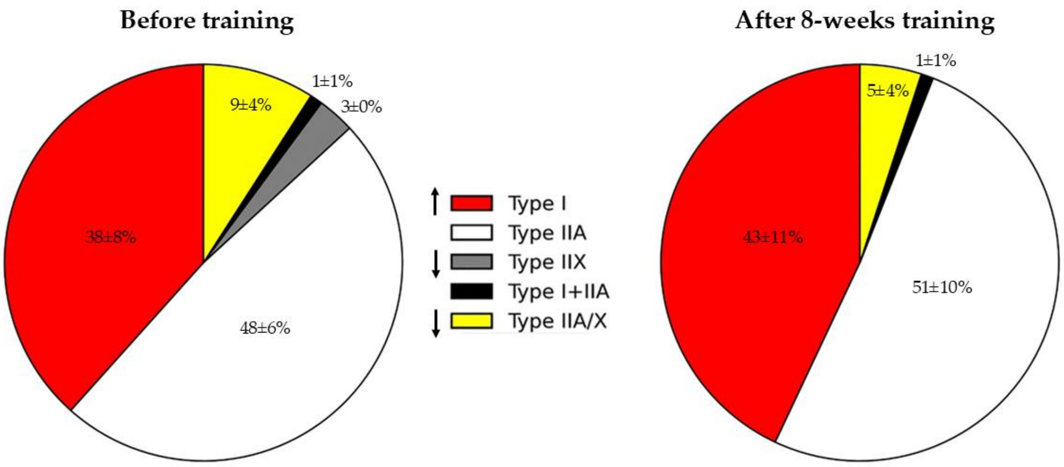

Figure S2. Muscle fibre-type distribution before and after 8 weeks of resistance training. The figure provides a descriptive overview of fibre-type distribution (mean ± SD) before and after 8 weeks of resistance training in all participants (n = 17), illustrating a shift toward a more oxidative profile. Statistical analyses are presented in the main text.
